# Immunomodulatory Effects of *Streptococcus suis* Capsule Type on Human Dendritic Cell Responses, Phagocytosis and Intracellular Survival

**DOI:** 10.1371/journal.pone.0035849

**Published:** 2012-04-27

**Authors:** Marjolein Meijerink, Maria Laura Ferrando, Geraldine Lammers, Nico Taverne, Hilde E. Smith, Jerry M. Wells

**Affiliations:** 1 Host-Microbe Interactomics, Animal Sciences, Wageningen University, Wageningen, The Netherlands; 2 Animal Sciences Group, Wageningen University, Lelystad, The Netherlands; Louisiana State University, United States of America

## Abstract

*Streptococcus suis* is a major porcine pathogen of significant commercial importance worldwide and an emerging zoonotic pathogen of humans. Given the important sentinel role of mucosal dendritic cells and their importance in induction of T cell responses we investigated the effect of different *S. suis* serotype strains and an isogenic capsule mutant of serotype 2 on the maturation, activation and expression of IL-10, IL-12p70 and TNF-α in human monocyte-derived dendritic cells. Additionally, we compared phagocytosis levels and bacterial survival after internalization. The capsule of serotype 2, the most common serotype associated with infection in humans and pigs, was highly anti-phagocytic and modulated the IL-10/IL-12 and IL-10/TNF-α cytokine production in favor of a more anti-inflammatory profile compared to other serotypes. This may have consequences for the induction of effective immunity to *S. suis* serotype 2 in humans. A shielding effect of the capsule on innate Toll-like receptor signaling was also demonstrated. Furthermore, we showed that 24 h after phagocytosis, significant numbers of viable intracellular *S. suis* were still present intracellularly. This may contribute to the dissemination of *S. suis* in the body.

## Introduction

*Streptococcus suis* is a major pathogen of swine, causing considerable economic losses and animal health care problems for the pig farming industry worldwide [1]. The natural habitat of *S. suis* is the upper respiratory tract and the intestinal tract [2], [3]. In adult pigs carriage of *S. suis* is usually asymptomatic but colonized sows can infect their piglets after nasal or oral contact [4]. Newborn pigs can also become infected during parturition when they contact, swallow or aspirate *S. suis* from sow vaginal secretions [5]. In young pigs *S. suis* infection causes a wide variety of diseases, including meningitis, septicemia which are the main causes of mortality. *S. suis* is also emerging as a serious zoonotic pathogen of humans particularly in South East and East Asia where it is one of the most common causes of human meningitis [6], [7]. In 2005 a large outbreak of 215 cases *S. suis* infections occurred in Sichuan, China, resulting in 38 deaths [8]. There are 33 serotypes of S. *suis* of which serotype 2 is most commonly associated with disease in humans and pigs worldwide [9], [10]. In addition serotypes 1 to 9 and 14 are responsible for infections in pigs [11] and serotypes 1, 4, 5, 14, 16 and 24 have caused severe disease in a limited number of persons [12], [13], [14], [15]. The capsule is known to be a very important virulence factor in *S. suis* [16]although not all capsulated isolates (including serotype 2) are virulent, highlighting the importance of other virulence factors in the pathogenesis of disease [3].

Dendritic cells (DCs) are important sentinels in the skin and mucosal surfaces that contact the external environment and play a key role in the homoeostatic control tolerance and immunity in the mucosal tissues [17]. Stromal factors such as retinoic acid and thymic stromal lymphopoietin imprint tolerogenic properties on resident DC. However when invading microbes are encountered the homeostatic mechanism are overridden by chemotactic recruitment of DC and their activation by pattern recognition receptor (PRR) binding to pathogen-associated molecular patterns (PAMPs). Upon activation DCs express up to 100x more MHC than monocytes, macrophages and neutrophils other antigen presenting cells (APCs) and migrate to mucosal associated lymphoid tissue to induce antigen-specific T cell responses [18], [19]. Thus DCs are instrumental in the orchestration of adaptive immune responses. Cytokines produced by activated DC have a major influence on T cell polarization, differentiation and clonal expansion. Interleukin (IL)-12 and tumor necrosis factor (TNF)-α, are pro-inflammatory cytokines that promote T helper (Th) 1 cell responses, whereas IL-10 is an anti-inflammatory cytokine that can promote induction of Th2 cells or regulatory T cells depending on the expression of other tolerizing factors [20].

DCs recognize different types of PAMPs using pattern recognition receptors (PRRs) of the Toll-like receptor (TLR), nucleotide-binding oligomerisation domain receptor (NLR) and C- type lectin receptor (CLR) protein families, [17], [21], [22]. PRR signaling is critical to DC maturation and in recent years much emphasis has been given to dissecting the innate signaling pathways involved in pathogen recognition. Each PRR recognizes variants of a specific molecular pattern and can be expressed on the cell surface, in intracellular compartments or in the cytosol. TLR1, 2, 4, 5, 6 and 11 recognize mainly microbial envelope components and are expressed on the cell surface, TLR3, 7, 8 and 9 recognize microbial nucleic acids and are expressed in intracellular compartments such as the endoplasmic reticulum, endosome and phagosome. TLR2 can form heterodimers with TLR1 or TLR6 to detect different, but related ligands. TLR2/1 recognizes tri-acyl lipoproteins found predominantly in Gram-negative bacteria and TLR2/6 the diacyl groups on lipoteichoic acid and lipoproteins of Gram-positive bacteria. NOD1 and NOD2 are cytoplasmic receptors that can detect peptidoglycan fragments produced in the phagosome or phagolysosome of antigen presenting cells although the nature of the transporters involved in translocation to the cytoplasm remains unknown [23]. The CLR family is characterized by the presence of one or more C-type lectin-like domains (CTLDs) and bind mainly sugars including self-antigens. CLRs trigger distinct signaling pathways that induce the expression of specific cytokines which determine T cell polarization fates [24].

Recently the interactions of a virulent serotype 2 strain and its unencapsulated derivative with porcine DC were studied *in vitro*. The capsular polysaccharide was shown to interfere with phagocytosis and consequently the level of DC maturation and production of several cytokines was reduced compared to an unencapsulated strain [25]. Given the emergence of *S. suis* as a significant cause of meningitis in humans we investigated the effect of different serotypes (SS1, SS2, SS4, SS7, SS9 and SS14) and the unencapsulated mutant of *S. suis* serotype 2 (SS2 J28) on the maturation and expression of IL-10, IL-12p70 TNF-α in human monocyte-derived DC. Additionally, we compared the efficiency of the different isolates in DC phagocytosis assays and studied the intracellular survival of internalized *S. suis* serotype 2of internalized *S. suis* serotype 2 S10 and its unencapsulated isogenic mutant. The ability of the different serotype strains to induce TLR signaling via human TLR2/6 was also investigated using a TLR2/6 specific luciferase reporter cell line. To our knowledge this is the first study concerning the interactions of *S. suis* with human DC and it provides new knowledge of the role of different capsular polysaccharide serotypes in the avoidance of host innate immunity.

## Materials and Methods

### Bacterial strains

Six different serotypes (SS1, SS2, SS4, SS7, SS9 and SS14) and the unencapsulated mutant of SS2 (SS2 J28) were obtained from Central Veterinary Institute, Lelystad NL (Table 1). In table 1 for each strain the expression of three virulence markers are indicated: two secreted cell wall located proteins namely the muramidase-released protein (MRP) and the extracellular factor (EF) [26], [27], and secreted hemolytic toxin suilysin (SLY) [28]. MRP and EF variants have been designated as MRP^S^ and EF*. All *S. suis* strains were cultured overnight at 37°C in Todd Hewitt broth (Oxoid). The bacteria were then recovered by centrifugation, washed twice in phosphate buffered saline (PBS, pH = 7.4), resuspended at approximately 1×10^9^ colony forming units (CFU)/mL in PBS containing 20% glycerol, and stored in aliquots at −80°C prior to use. The exact number of bacterial CFU in a thawed aliquot was determined by plating serial dilutions on Columbia blood agar plates (BD) containing 5% sheep blood in presence of 5% CO_2_.

**Table 1:** List of strains used in this study.

Serotype	Strain	Virulence for pigs	MRP	EF	Suilysin	CPS	Reference
SS1	6388	HV	MRP^s^	EF^+^	SLY^+^	Cps1^+^	[47], [48]
SS2	S10	V	MRP^+^	EF^+^	SLY^+^	Cps2^+^	[16]
SS2 J28	10cpsΔEF^a^	AV	MRP^+^	EF^+^	SLY^+^	Cps2^−^	[16]
SS4	5213	ND	MRP^s^	–	ND	Cps4^+^	[49]
SS7	8039	ND	–	–	–	Cps7^+^	[48], [50]
SS9	8067	AV	–	–	SLY^+^	Cps9^+^	[48], [51]
SS14	13730	ND	–	EF*	ND	Cps14^+^	–

a The isogenic unencapsulated mutant strain 10 cpsΔEF parts of the cps2E and cps2F gene were replaced by an antibiotic resistance gene. HV high virulent V virulent AV avirulent. MRP muraminidase-released protein. EF extracellular factor. SLY suilysin. CPS capsular polysaccharide synthesis *, higher MW protein expressed; s, smaller MW protein expressed.

### Differentiation and maturation of dendritic cells

The study was approved by the Wageningen University Ethical Committee and was performed according to the principles of the Declaration of Helsinki. Buffy coats from four blood donors were obtained from the Sanquin Blood bank Nijmegen, Netherlands. A written informed consent was obtained before the sample collection. Human monocytes were isolated from blood using a combination of Ficoll density centrifugation and cell separation using CD14-specific antibody coated magnetic microbeads (Miltenyi Biotec). The purity of isolated CD14+ cell fraction was greater than 90% and viability >95% in all experiments. To generate immature DC (iDCs), the purified CD14+ cells were cultured for 6 days in RPMI 1640 medium (Invitrogen), supplemented with 100 units/mL penicillin G (Invitrogen), 100 µg/mL streptomycin (Invitrogen), IL-4 (R&D systems) and granulocyte-macrophage colony-stimulating-factor (GM-CSF) (R&D systems). GM-CSF and IL-4 were added to differentiate the monocytes into myeloid DCs. At day 6 the iDCs (1×10^6^ /mL) were stimulated with LPS (1 µg/mL) or the different *S. suis* serotypes at multiplicities of infection (MOI) of 1 bacterium per DC or 10 bacteria per DC for 48 hours. Unstimulated iDCs were used as a negative control.

### Analysis of cell surface markers and measurement of cell death by flow cytometry

During the 8 day culture period of the CD14+ cells (6 days of differentiation of monocytes into immature dendritic cells and two days of stimulation), cells were stained on days 3, 6 and 8 with fluorescence-conjugated monoclonal antibodies specific for CD83, CD86 or their isotype-matched controls (BD biosciences, San Diego, USA) and analyzed by flow cytometry (FACSCanto II, BD, San Diego, USA) to check the maturation and activation status of the cells. CD86 and CD83 were not expressed on immature dendritic cells (d 3 and 6) but were highly expressed on DCs after activation with known maturation factors (e.g. LPS). The magnitude of the response from different human donors can vary considerably so for comparison the data was normalized to the LPS control sample data (100%) for each donor.

On days 3, 6 and 8 the percentage of viable cells was measured by flow cytometry (FACSCanto II, BD, San Diego, USA). Live, apoptotic and necrotic cells were discriminated by staining with Annexin V and propidium iodide on days 3, 6 and 8 according to the manufacturer's protocol. The cells were analyzed on a flow cytometer (FACSCanto II, BD, San Diego, USA). Cells that are negative for both Annexin V and PI are not apoptotic or necrotic as translocation of the membrane phospholipid phosphatidylserine has not occurred and the plasma membrane is still intact. Therefore, Annexin V and PI double negative cells were considered as viable cells, whereas both single and double positive cells were regarded as non-viable [29]. The flow cytometry data was analyzed using the BD FACSDiva software. On days 3 to 8 the viability of the cells was between 60 and 95%. There were no significant differences in cell death between *S. suis* co-cultures or compared to the medium and LPS controls.

### Cytokine assay

Supernatants from the DC stimulation assays were collected after stimulation for 48 hours, and analyzed for the presence of cytokines (IL-10, IL-12p70 and TNF-α) using a cytometric bead-based immunoassay that enables multiplex measurements of soluble cytokines in the same sample [30], according to the manufacturer's protocol (BD biosciences). The limits of sensitivity for detection were as follows: 0.13 pg/mL, 0.6 pg/mL and 0.7 pg/mL. The flow cytometry data were analyzed using the BD FCAP software.

### Phagocytosis assay

The iDCs (10^6^ cells) were inoculated with the different *S. suis* serotypes (MOI 10) and incubated for one hour in antibiotic-free RPMI 1640 at 37°C and the presence of 5% CO_2_. The DCs were further incubated for one hour in RPMI 1640 containing 56.2 µg/mL penicillin G and 100 µg/mL gentamicin. Subsequently the DCs were collected and the centrifuged for 5 minutes at 845 g. The pellet was washed with PBS to remove the antibiotics and the DCs lysed and vigorously vortex in ice-cold milliQ water. The cell lysate was then serial plated on Columbia blood agar plates (BD) containing 5% sheep blood to enumerate the CFU of *S. suis*.

#### Adhesion and Phagocytosis Assay

The iDCs (10^6^ cells) were inoculated with SS2 and SS2 J28 (MOI10) and incubated for 1 hour in antibiotic-free RPMI1640. To count the adherent and phagocytosed bacteria DCs were washed after one hour twice with PBS to remove the unbound bacteria, lysed with ice-cold milliQ water and plated on Columbia blood agar plates (BD) containing 5% sheep blood.

### Kill Curve

Phagocytosis of *S. suis* was performed as described above and then the DCs were incubated in RPMI 1640 containing 56.2 µg/mL penicillin G and 100 µg/mL gentamicin to kill extracellular bacteria. The killing of phagocytosed *S. suis* was determined after 1, 2, 3 and 4 h by removing the antibiotics with PBS washes, lysis in ice-cold milliQ water and serial plating on Columbia blood agar plates (BD) containing 5% sheep blood.

### Survival of *S. suis* inside DCs after 2 and 24 hours

The iDCs (10^6^ cells) were inoculated with SS2 and SS2 J28 (MOI10) and incubated for one hour in antibiotic-free RPMI1640. After one hour of incubation antibiotics (100 µg/mL gentamicin and 56.2 µg/mL of penicillin G) were added to kill all the extracellular bacteria. After a further one hour incubation in the presence of the antibiotics DCs samples were collected and plated in the same way as described in the phagocytosis assay (2 h time point). After a further 4 hours, the medium was replaced by RPMI lacking antibiotics to prevent the antibiotics from entering the DCs and killing intracellular bacteria. After a total of 23 hours incubation the DCs were incubated for one hour in RPMI with or without antibiotics washed twice with PBS, lysed with ice-cold milliQ water and plated on Columbia blood agar plates (BD) containing 5% sheep blood (24 h time point).

### TLR2/6 assay

The TLR2/6 signaling assay was performed essentially as previously described [31]. Briefly, HEK293 cells (Invivogen, Toulouse, France) were transformed with human TLR2/6 and pNIFTY, a NF-κB luciferase reporter construct (Invivogen). The cells were plated a concentration of 6×10^4^ cells per well in DMEM medium. Cells were then stimulated with the different *S. suis* strains, Pam2CSK as a positive control and with medium alone (negative control) and incubated at 37°C and 5% CO_2_ for 24 hours. After this incubation period the medium was replaced with Bright glow (Promega), the plate was vortexed and the luminescence was measured using a Spectramax M5 (Molecular Devices). Human embryonic kidney (HEK)293 cells not expressing TLR receptors but harbouring pNIFTY, a NF-κB luciferase reporter construct (Invivogen, Toulouse, France) were used as the negative control in the NF-κB assays.

### Electron microscopy

For morphological analysis of the capsule structure, samples of exponential phase (∼0.5 _OD600_) bacteria were fixed according to the lysine-acetate-based formaldehyde/glutaraldehyde ruthenium red-osmium (LRR) fixation procedure, as described previously [32] and studied by JEOL JEM 2100 transmission electron microscope at magnifications of 25.000 X.

### Statistical analysis

Dixon's Q test was applied for the evaluation of differences in the values of the immune- and cytokine assays. Datasets contained values of six different donors. P values of <0.05 were considered significant.

Independent sample t-test was applied for the evaluation of differences between SS2 and SS2J28 in the phagocytosis assay and the kill curve. P<0.05 were considered significant.

## Results

### *S. suis* capsule serotype differentially affects DC maturation and activation

**Figure 1:**
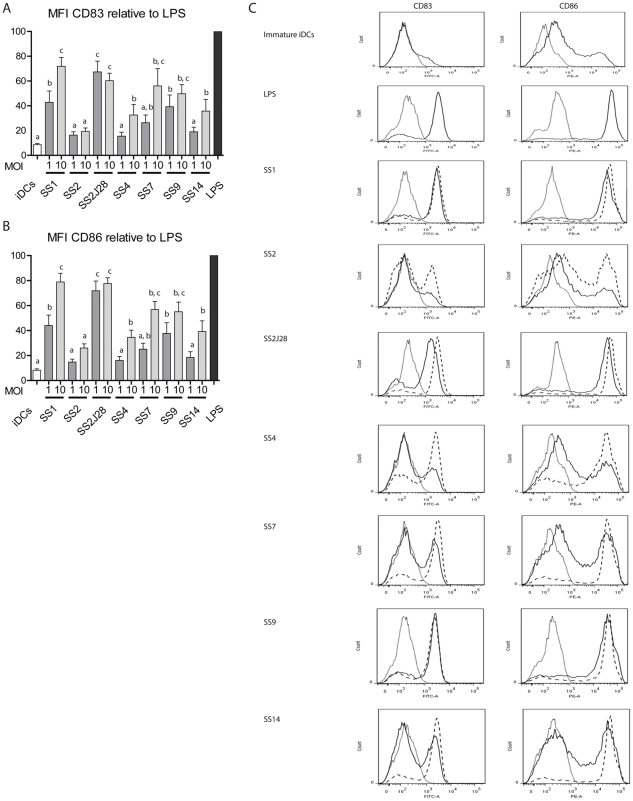
Mean Fluorescence Intensity (MFI) of dendritic cells normalized with LPS. The MFI of stained cell surface markers by monocyte derived dendritic cells with 6 different *S. suis* strains and SS2J28 mutant, with immature DCs as the negative control and LPS as the positive control. **A.** MFI of CD83 **B.** MFI of CD86. Bars showing unequal letters significantly differ in their surface marker expression (P<0.05). **C.** Histograms for expression of surface markers CD83 and CD86. Dotted lines represent the isotype controls and black lines the stimulated samples. In case of bacteria a black line represents a MOI 1 and a dashed line a MOI 10.

Immature monocyte-derived DCs derived from six different human donors were used as *in vitro* model to investigate interactions with *S. suis*. The DCs were stimulated for 48 hours with 6 different *S. suis* serotypes and SS2J28 at MOI 1 and MOI 10 (Fig. 1). Expression of the surface expressed co-stimulatory molecule CD86 and maturation marker CD83 were measured to determine the activation and maturation status of the DCs respectively (Fig. 1A and B for mean fluorescence intensity and 1C for histograms). For all encapsulated strains stimulation of DC with *S. suis* at a MOI 10 resulted in higher maturation and activation marker expression than at MOI 1. The induction of the surface expression CD86 and CD83 differed markedly among the capsule serotypes tested. Significantly higher levels of CD83 and CD86 were observed following DC stimulation with serotypes SS1, SS7 and SS9 and the unencapsulated SS2 mutant than with serotype SS2. Interestingly, serotype SS2 was the least effective at maturating DC although its unencapsulated variant, SS2J28 was the most effective indicating the importance of the capsule in the avoidance of host innate immunity.

### The capsule of *S. suis* serotype 2 differentially modulates the IL-10 to IL-12 ratio

**Figure 2:**
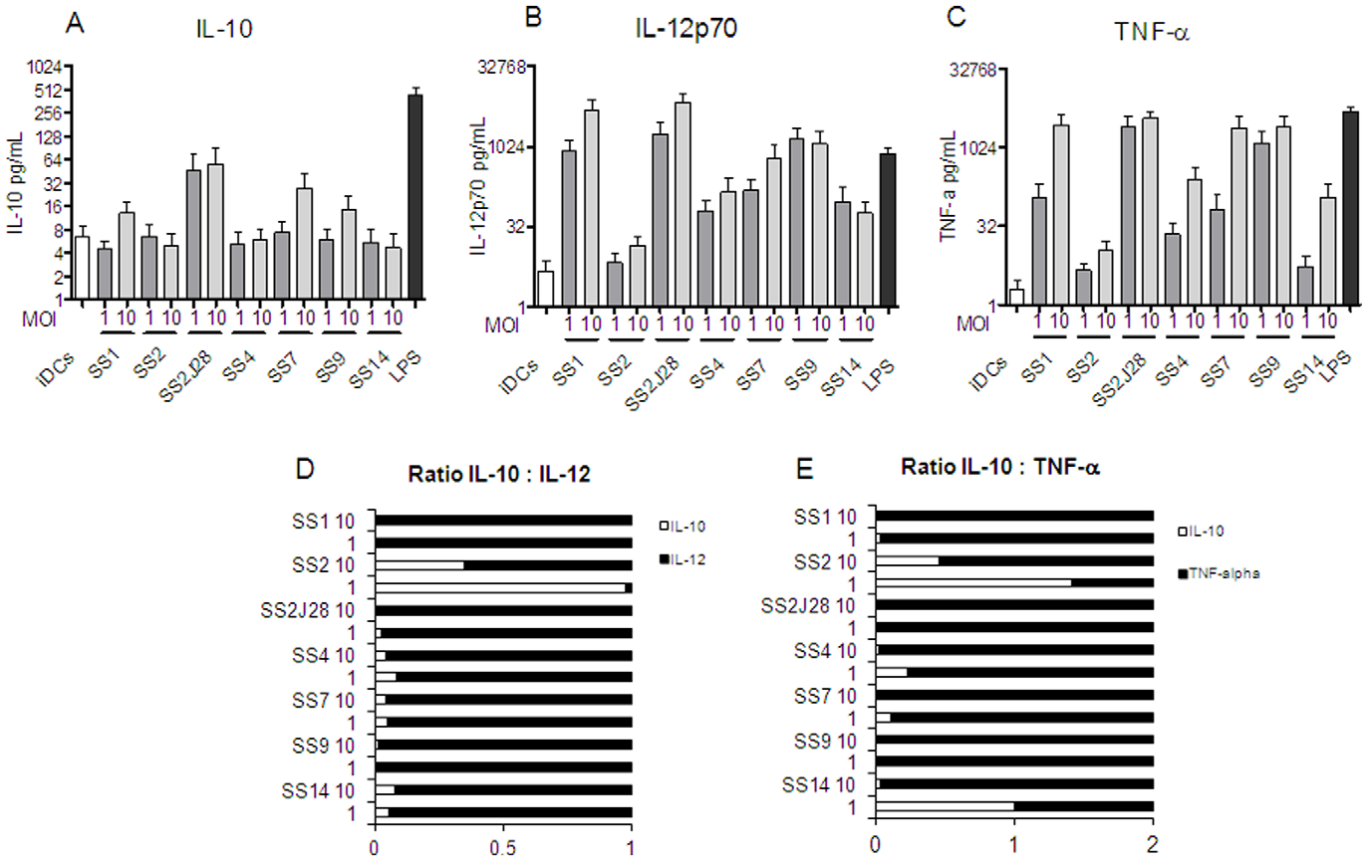
Cytokine secretion by dendritic cells. Cytokine production by monocyte derived dendritic cells with 6 different *S. suis* strains and SS2J28, with immature DCs as the negative control and LPS as the positive control. **A.** IL-10 **B.** IL-12p70 **C.** TNF-α. **D.** IL-10/IL-12 ratio. **E.** IL-10/TNF-α.

The amounts of IL-10, IL-12 and TNF-α measured in the supernatants of DC co-cultured with the different serotypes was highly variable (Fig. 2). The amounts of IL-10 ranged from 5 pg/mL to 56 pg/mL, IL-12p70 from 7 pg/mL to 6948 pg/mL and TNF-α from 5 pg/mL to 3744 pg/mL (Fig. 2A–C). As expected, stimulation with an MOI 10 resulted in higher amounts of secreted cytokine than stimulation with an MOI 1. In keeping with the data on maturation markers (Fig. 1) serotypes SS1, SS7 and SS9 were the highest inducers of cytokines, whereas serotype SS2 induced the lowest amounts of cytokines (all cytokine <10 pg/mL). In contrast the unencapsulated derivative SS2J28 stimulated the highest amounts of IL-10 and IL-12 and high amounts of TNF-α. The ratio of IL-10 to IL-12 is often used as an indicator of the potential to polarize T cell responses towards Th1 or Th2/Treg [17]. Interestingly all of the *S suis* serotypes except SS2 induce low IL-10 to IL12 ratios (less than 0.08). For SS2 the IL-10 to IL-12 ratio was 0.34 at MOI 10 and almost 1.0 (0.98) at MOI 1. Strikingly, the unencapsulated derivative of SS2 designated SS2J28 has a much lower IL-10 to IL-12 ratio than SS2 (0.03 at MOI 1 and 0.008 at MOI 10) suggesting that the type 2 capsule can down-regulate the host cell-mediated response to *S. suis* (Fig. 2D). Similar trend of ratio's were observed for the IL-10 to TNF-α ratio (Fig. 2E).

### Effect of capsular polysaccharide on the capacity of DCs to internalize *S. suis*

**Figure 3:**
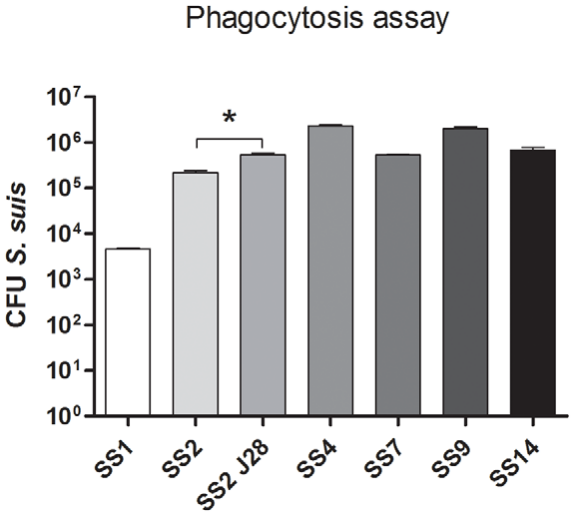
Phagocytosis assay. Phagocytosis by immature DCs with 6 different *S. suis* strains and an unencapsulated mutant at an MOI 10. This is a representative figure from 1 donor, out of 5 donors. *P<0.05.

The percentage of *S. suis* phagocytosed by DCs varied considerably among the different serotypes tested. After one hour of incubation of the DCs with the bacteria, SS4 and SS9 were more efficiently taken up by the DCs (respectively 23% and 20% of original inoculum (10^7^ bacteria) compared to the other strains (Fig. 3). In contrast capsule types SS1 and SS2 were relatively resistant to phagocytosis by DCs (0.04% and 2.16% respectively the original inoculum). The unencapsulated mutant was internalized at significantly higher amounts than its wild type SS2 progenitor (5.29% vs 2.16%; P = 0.0001). Surprisingly however, the unencapsulated strain was less efficiently internalized than serotype strains SS4 and SS9 (Fig. 3).

### Capsular type 2 does not affect intracellular survival of internalized *S. suis*

**Figure 4:**
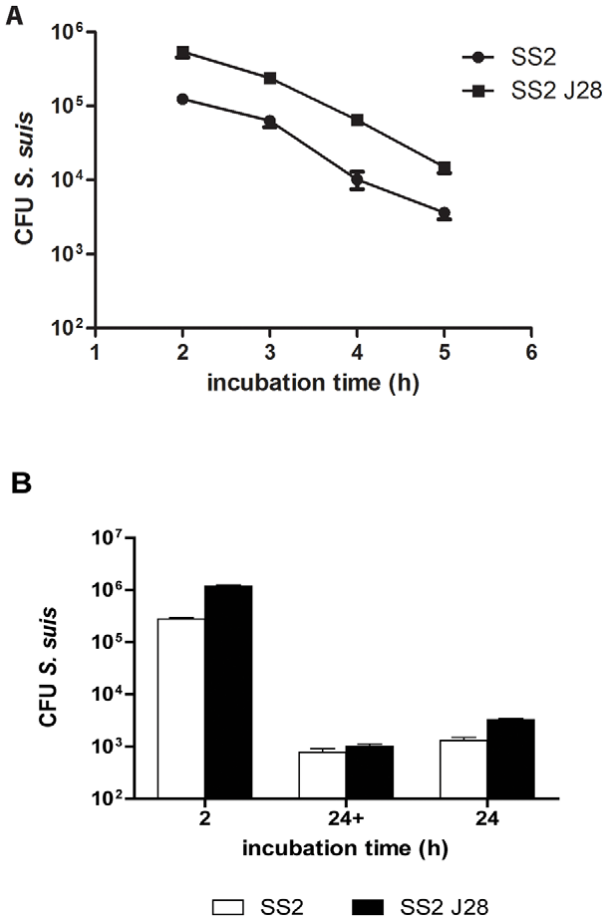
Survival of *S. suis* inside dendritic cells. **A.** *Kinetics of adhesion and phagocytosis* Immature DCs were inoculated with SS2 and SS2 J28 at a MOI of 10 bacteria/DC for subsequently 2, 3, 4, 5 and 24 hours of incubation. **B.** *Survival of S. suis inside DCs after 2 and 24* *hours* Immature DCs were inoculated with SS2 and SS2J28 at a MOI10 for 2 and 24 hours of incubation. The last hour the DCs were incubated with (24+) or without (24) antibiotics.

To rule out the possibility that differences in internalization of SS2 and SSJ28 by DC might be due to strain variation in intracellular survival we measured the survival of these two strains in DC over time. After one hour of incubation with iDC antibiotics were added for 1 hour to ensure that only internalized bacteria were counted after lysis of the DCs. After a total 2 hours of incubation the number of viable bacteria inside the DCs decreased considerably, to 39% of the original inoculum for SS2 and 43% of the inoculum for SS2J28 (Fig. 4A). Over the first 4 hours the number of viable *S. suis* decreased at a similar rate for both strains indicating that the higher level of internalization measured for SS2 J28 (Fig. 4A) could not be due to less rapid killing.

### Viable *S. suis* reside within DC 24 hours after phagocytosis

To examine the survival of the wild type SS2 and its mutant SS2J28 inside the DCs after 24 hours, the DCs (10^6^ cells) and bacteria (MOI 10) were incubated for 2 and 24 hours (Fig. 4B). After 1 hour of incubation antibiotics were added to the medium kill extracellular and adhered bacteria. After 5 hours, the medium was replaced by RPMI without antibiotics, to prevent the antibiotics from entering the DCs. After 24 hours around 10^3^ CFU/mL of live *S. suis* were recovered from the lysed DC suggesting that a small proportion of the bacteria could survive intracellularly. To rule out that these phagocytosed bacteria were released from DC after 5 hours of co-incubation and were growing in the medium the experiment was repeated using an additional antibiotic treatment at 23 hours to kill any extracellular/adherent bacteria that might be present. The results (Fig. 4B) showed that the CFU/mL counts present after 24 hour could be attributed to the presence of intracellular *S. suis*.

### Involvement of TLR2 and TLR6 in innate immune signaling by *S. suis*

**Figure 5:**
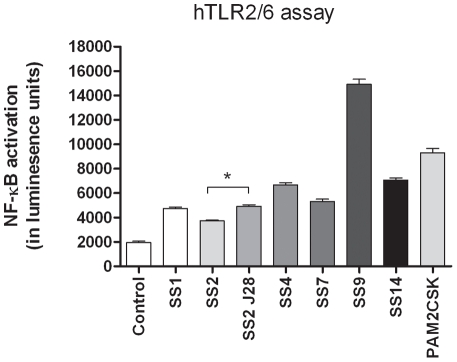
hTLR2/6 assay. HEK293 cells were incubated with 6 different *S. suis* strains and an unencapsulated mutant at a MOI10, PAM2CSK as a positive control and medium as a negative control. This figure is representative out of three hTLR2/6 assays. *P<0.05.

TLR2/6- mediated activation of NF-κB could be one of the major pathways for DC activation via LTA or lipoproteins in the cell envelope of *S. suis*. Therefore we tested the TLR2/6 signaling capacities of all the serotypes in a reporter assay using HEK293 cells expressing human TLR2 and TLR6 heterodimer that recognizes lipoteichoic acid (LTA) and lipoprotein lipid anchors in Gram-positive bacteria (Fig. 5). HEK293 cells transformed with only the pNIFTY, a NF-κB luciferase reporter construct did not respond to Pam_2_CSK demonstrating the dependency of NF-κB activation on co-expression of hTLR2/6 receptor. Medium was used as a negative control and Pam_2_CSK (synthetic agonist of TLR2/6) as a positive control. The results shown in Fig 5 demonstrate that indeed all strains are capable of triggering NF-κB activation via TLR2/6 signaling but there was no correlation between the capacity of the strains to induce TLR2/6 signaling and activate DC (Fig. 2). Interestingly the unencapsulated mutant of SS2 induced significantly (P<0.05) higher levels of NF-κB than SS2 indicating that the capsule has a shielding effect on TLR activation.

### Morphological analysis of SS2 and SS2J28

**Figure 6:**
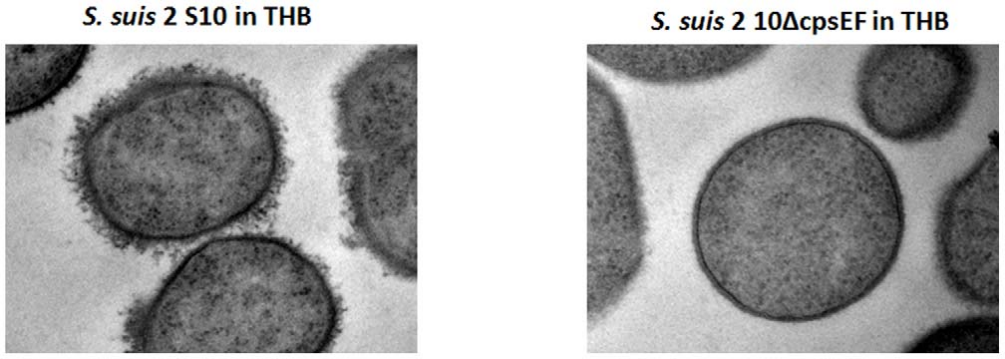
Detection of encapsulation of *S. suis* strains by LRR staining and transmission electron microscopy. *S. suis* 2 strain 10 shows a thick capsule, whereas no capsular material can be seen in isogenic mutant strain *S. suis* 2 10ΔcpsEF.

For morphological analysis of the capsule structure, samples of exponential phase (∼0.5 _OD600_) SS2 and SS2J28 were studied by electron microscope at magnifications of 25.000 X. As expected SS2 showed a thick capsule whereas no capsular material can be observed in the isogenic mutant strain SS2J28 (Fig. 6).

## Discussion

Apart from its major economic impact on the mortality and morbidity of young pigs in agro-food production *S. suis* is emerging as a one of the major causes of meningitis in South East Asia. To gain further knowledge on the role of *S. suis* capsule and capsule serotype on the immune response of the human host we compared phagocytosis and immune responses of human immature DC to different serotypes of *S. suis*. DCs are professional antigen presenting cells that play a key role in the induction of adaptive immune responses. Once activated by contact with invading pathogens CD103^+^ DC can traffic from the sites of mucosal infection to the draining lymph nodes to induce T cell responses. Additionally they play a crucial role in induction of adaptive immune responses in the Peyer's patches of the nasal mucosa and small intestine. Many pathogens have evolved mechanisms to avoid phagocytosis including production of leukotoxins, the inhibition of complement activation and masking of binding sites for endocytic receptors by polysaccharide capsules [33].

The serotypes of *S. suis* differed significantly in their ability to activate DCs and induce cytokine responses. Serotypes SS1, SS7 and SS9 induced expression of significantly higher levels of DC activation and maturation markers than serotypes SS2, SS4 and SS14 (Fig. 1). Interestingly SS2, the strain that was the least effective at activating and maturating DC was serotype 2, which is the serotype most commonly associated with invasive disease in pigs and humans [34]. In contrast the unencapsulated variant of SS2 (SS2J28) was the most effective strain in maturating and activating DC, indicating the important role of the capsule in shielding cell wall components that activate DC and induce cytokine responses (Fig. 1). The thickness of the capsule may also influence the activation by influencing the release of MAMPs such as lipoproteins and LTA. Capsule serotype 1 has been reported to have a thinner capsule than other serotypes [51] and interestingly this was the most effective of all capsulated strains in activating and maturating DC. The anti-phagocytic effects of SS2 capsule were apparent from the significantly higher phagocytosis of the unencapsulated mutant SS2J28 (5.29% vs 2.16%; P = 0.0001) (Fig. 3). To rule out the possibility that differences in internalization of SS2 and SS2J28 by DC might be due to strain variation in intracellular survival we measured survival over period of 4 hours after phagocytosis. The number of viable *S. suis* decreased at a similar rate for both strains indicating that the higher level of internalization measured for the unencapsulated mutant SS2J28 (Fig. 3) was not due to less rapid killing (Fig. 4A). Similar results were recently described using porcine DCs and a capsulated and unencapsulated serotype 2 strain [25]. As in our own study an unencapsulated mutant of a serotype 2 strain was phagocytosed at significantly higher levels and once internalized, both the wild-type strain and its non-encapsulated mutant were killed at similar rates.

To investigate the effect of other capsule types on phagocytosis the internalization of several other serotypes were compared to serotype 2 and its unencapsulated mutant SS2J28 (Fig. 3). The serotypes differed considerably in their ability to be phagocytosed with around 20% of the inoculum being internalized in the case of SS4 and SS9 but only 2% in the case of SS2 (Fig. 3). This might be explained by the difference in composition of the capsulesand their charge which is known to be important in the avoidance of phagocytosis. A recent genetic analysis of the capsular polysaccharide synthesis locus of 15 *S. suis* serotypes predicted that capsules of serotypes 1, 2, and 14 may contain sialic acid [35]. In *Streptococcus agalactiae* capsule sialic acid has been shown to increase the hydrophilic surface properties of the bacteria and have an inhibitory effect on phagocytosis [36]. This might be an explanation for the fact that phagocytosis of serotype strains 1, 2 and 14 was significantly lower than for serotype strains 4 and 9. The sialylated capsule of *Streptococcus agalactiae* also inhibits C3 deposition on the bacterial cell surface [37], probably via recruitment of factor H, an anti-activator of the complement alternative pathway [38]. However it is not evident that C3 deposition is inhibited by sialic acid in the serotype 2 capsule of *S. suis* because phagocytosis levels are not significantly different in the presence or absence of serum factors [25].

Interestingly the unencapsulated SS2J28 strain was less efficiently phagocytosed than serotype strains SS4 and SS9 (Fig. 3). Thus it is possible that SS2 has other mechanisms that inhibit phagocytosis. A two-component regulatory system (TCS) designated SalK/SalR has been shown to have a protective role in the killing of *S. suis* by granulocytes [16], [39], [40] but this locus is absent in the genome of SS2 strain and thus cannot be responsible for the lower levels of phagocytosis observed for SS2 compared to SS4, SS7, SS9 and SS14.

A shielding effect of SS2 capsule was investigated using a reporter cell line for TLR2/6 signaling. The TLR2/6 heterodimer is formed by binding of the di-acyl groups present on lipoproteins of Gram-positive bacteria and lipoteichoic acids present in the cell wall [22], [41]. This triggers NF-κB activation via a signal kinase cascade involving the adapter protein MyD88 and was detected in our assay by production of luciferase under control of an NF-κB promoter. The unencapsulated mutant of SS2 induced significantly (P<0.05) higher levels of NF-κB than SS2 suggesting that the capsule has a shielding effect on the exposure of TLR agonists that can activate DCs (Fig. 5). Notably, the level of NF-κB activation obtained with the unencapsulated mutant was significantly lower than for SS9 (P<0.0001), SS14 (P<0.0001) and SS4 (P<0.0001). The highest level of TLR2/6 activation was observed for SS9, something observed in a previous study using the same strains SS2 and SS9 (Fig. 5) [42]. Interestingly we found that SS9 was phagocytized more efficiently than the other strains and was highly effective at activating DC. However efficiency of phagocytosis did not correlate with activation of DC as evident for strain SS1 which was phagocytized at relatively low levels compared to the other serotypes but nevertheless strongly activated DC in co-culture.

The amounts of IL-10, IL-12, and TNF-α measured in the supernatants of DC co-cultured with the different serotypes was highly variable (Fig. 2). The amounts of IL-10 ranged from 5 pg/mL to 56 pg/mL, IL-12p70 from 7 pg/mL to 6948 pg/mL and TNF-α from 5 pg/mL to 3744 pg/mL (Fig. 2A–C). In agreement with the data on maturation and activation markers (Fig. 1) the serotypes SS1, SS7 and SS9 were the highest inducers of cytokines, whereas serotype SS2 induced the lowest amounts of cytokines (all cytokine <10 pg/mL). Interestingly all of the *S suis* serotypes except SS2 induce low ratios of IL-10 to IL12 ratios (less than 0.08). For SS2 the IL-10 to IL-12 ratio was 0.34 at MOI 1 and almost 1.0 (0.98) at MOI 10. This qualitative effect on the cytokine response was due to the serotype 2 capsule because the unencapsulated mutant of SS2 induced cytokines with a low IL-10 to IL-12 ratio (0.03 at MOI 1 and 0.008 at MOI 10) as observed for the other serotypes (Fig. 2D). A similar trend was seen for the IL-10/TNF-α cytokine ratio (Fig. 2E). Although induced levels of IL-10 were relatively low for both strains the unencapsulated mutant induced more than 100 fold higher levels of the pro-inflammatory IL-12 and TNF-α than the capsulated strain. It is not known whether the immunomodulatory effect of SS2 capsule is also observed with porcine DC as the recent study did not measure IL-10 production by DC [25]. A consequence of increased IL-10 production may be the polarization of T helper cell responses towards Th2 or Treg [43]. In pathogenic species of *Yersinia* for example, the secreted V antigen protein induces IL-10 in macrophages to evade the host's inflammatory response during infection [44]. Pathogens such as *Mycobacterium tuberculosis* and HIV target DC-SIGN on DC to escape immunity. Binding to DC-SIGN cause internalization but not subsequent antigen processing and induces IL-10 expression resulting in suppression of Th1 responses [45]. DC-SIGN binds glycans containing high mannose structures appear not to be present in the published structure of the serotype 2 capsular polysaccharide [46]. However, it is possible that the involvement of other C –type lectin receptors on DC or other glycan structures may be involved in the immunomodulatory effects of the SS2 capsule.

Over a period of 5 hours after internalization in DC the number of viable *S. suis* were reduced about 100 fold. The rate of killing and overall levels of intracellular survival of *S. suis* after 5 hours was higher than that reported previously using porcine DC [25]. This may have been due the use of a different serotype 2 strain and/or differences in killing capacity of human and pig DCs and warrants further study. Despite the fact that a high proportion of phagocytized *S. suis* were killed by DC in the first 5 hours of incubation we were able to recover around 10^3^ CFU of SS2 and the unencapsulated mutant after 24 hours incubation (Fig. 4B). In these experiments antibiotics were added to the medium for 5 hours to kill extracellular and adhered bacteria then the medium was replaced by RPMI without antibiotics, to prevent the antibiotics from entering the DCs. Prior to lysis antibiotics were added a second time to some of the samples to kill any extracellular bacteria that might have been released from DC. The results (Fig. 4B) showed that the CFU counts present after 24 hour could indeed be attributed to the presence of viable intracellular *S. suis*. This has important consequences for pathogenesis because activated DCs eventually undergo apoptosis and may release viable *S. suis*. As DC traffic from the mucosa travel via the bloodstream to lymphoid tissue such a mechanism may enable *S. suis* to rapidly disseminate in the body during invasive disease.
